# Let’s Get Digital: Digital Biomarkers for Understanding ADRD

**DOI:** 10.1093/geroni/igaf122.1950

**Published:** 2025-12-31

**Authors:** Jennifer Schrack, Adam Spira, Amal Wanigatunga

## Abstract

Digital biomarkers from wearable sensors are emerging as sensitive, novel predictors of cognitive health. A growing body of evidence links elements of sleep and physical activity with both current cognitive status and longer-term dementia risk. To this end, “digital biomarkers” from wearable sensor data may enhance our ability to better detect and understand changes in brain health through remote assessments. Yet, many aspects of digital data remain under-addressed, including how to best leverage the multi-dimensional time series data produced by most sensors into clinically meaningful metrics. This symposium will focus on quantifying and defining digital measures of physical activity and sleep, and their associations with various features of brain and cognitive health in the Atherosclerosis Risk in Communities (ARIC) Neurocognitive Study. Dr. Schrack will present evidence on the association of long-term cognitive trajectories with subsequent physical activity. Mr. Gao will the discuss how time of day activity varies across these cognitive trajectories. Dr. Dougherty will describe the use of compositional data analysis to investigate the interplay between daily active and sedentary behaviors and their association with measures of cortical thickness. Dr. Chamberlin will present evidence on interdaily variability of physical activity intensity and the rest-activity cycle with dementia risk. Finally, Dr. Spira will present the association of rest-activity rhythms with measures of brain volumes, white-matter hyperintensity volume, and the presence of infarcts and microbleeds. Collectively, these presentations will highlight novel pathways by which digital biomarkers can be used to shed novel insights into cognitive health and ADRD.

